# Targeting critical source areas for phosphorus losses: Evaluation with soil testing, farmers’ assessment and modelling

**DOI:** 10.1007/s13280-017-0935-5

**Published:** 2017-08-04

**Authors:** Faruk Djodjic, Helena Elmquist, Dennis Collentine

**Affiliations:** 1 Department of Aquatic Sciences and Assessment, Swedish University of Agricultural Sciences, P.O. Box 7050, 75007 Uppsala, Sweden; 2 Farming in Balance, Franzengatan 6, 105 33 Stockholm, Sweden; 3 Department of Soil and Environment, Swedish University of Agricultural Sciences, Box 7014, 75007 Uppsala, Sweden

**Keywords:** Eutrophication, Farm, High-resolution, Phosphorus, Site-specific, Targeting

## Abstract

Diffuse phosphorus (P) losses from arable land need to be reduced in a cost-efficient way, taking into account their temporal and spatial variability. This study, based on 16 farms across southern Sweden, examined possibilities for identifying critical source areas for P losses based on the combined results of high-resolution erosion modelling, independent risk assessments by farmers, soil survey and SWOT analysis performed by farmers. Statistically significant differences in dissolved P release were found between soil P test classes in the studied area, whereas soil textural classes and not P content governed potential mobilisation of soil particles and unreactive P. Spatial comparison of problem areas identified by farmers and modelled features showed that the modelled erosion pathways intersected 109 in a total of 128 (85%) observed problem areas. The study demonstrates the value in involving farmers in the identification of critical source areas in order to select and support implementation of effective countermeasures.

## Introduction

Human activities, including modern agriculture, distort nitrogen (N) and phosphorus (P) flows and alter the status of lake and marine ecosystems (Rockstrom et al. 2009). Following the successful targeting of nutrient point sources such as municipal wastewater treatment plants, high diffuse losses from agricultural fields still remain to be addressed to reduce eutrophication of water recipients (HELCOM 2013). Achieving good ecological status for inland waters in line with the EU Water Framework Directive (WFD) as well as the ambitious Country Allocated Reduction Targets (CARTs) for the Baltic Sea agreed on at the HELCOM Copenhagen Ministerial Meeting (HELCOM 2013) will require measures that reduce the loads from agricultural practices.

The majority (~80%) of P losses originate from a small proportion of catchment areas (~20%), a situation known as the 80:20 rule (Sharpley et al. 2009). These so-called critical source areas (CSAs) coincide with hydrologically active, interconnected areas where overland and/or shallow subsurface flow mobilise and transfer P from terrestrial to aquatic ecosystems (Pionke et al. 2000). These CSAs vary spatially across individual watersheds and sometimes even within individual fields. As a result, effective measures to reduce P losses will require differing levels of management that are appropriate for different areas of farmland within individual watersheds (Gburek et al. 2000).

However, in spite of the extensive body of scientific evidence suggesting that P losses are episodic and spatially variable, current environment protection programmes are not designed to target the most vulnerable parts of the landscape but applied in a rather general way. At best, targeting efforts may include identification of fields with a high soil P content, in an attempt to address the source part of the P transfer continuum (Haygarth et al. 2005). From a farmer’s point of view, identifying CSAs and associated transport pathways might represent a win–win situation, as addressing nutrient losses within these limited areas may be more cost effective and at the same time allow for more intense production on non-sensitive parts of the farm. Agronomic soil P extraction tests offer one source of information for identifying CSAs (Sims 1998). Combining soil tests with other sources of information could even further contribute to more detailed identification of CSAs and at-risk areas within fields and support development of effective management plans. Additionally, modelling water flow pathways at catchment scale using digital elevation models (DEMs) and GIS-based soil hydrology classifications offers a useful template to identify and rank vulnerability to erosion and overland flow (Sharpley et al. 2015).

Recent developments in terms of accessibility to high-resolution data and modelling approaches have enabled accurate identification of CSAs at landscape and catchment scales. Djodjic and Villa (2015) were able to identify 72–96% of observed erosion and overland flow features in four different catchments using distributed modelling with high-resolution DEM, using only the top 2% of erosion-prone cells (2m × 2 m). Thomas et al. (2016) using an index to identify CSAs reported that 1.1–5.6% of the four catchment areas evaluated had the highest risk of legacy soil P transfers. While these approaches offer some advantages to, or in combination with, soil testing for identifying CSAs, transport pathways and appropriate management plans, a significant challenge remains. In order for this type of modelling and soil testing to be used at an individual farm level, policy makers and farmers must be convinced that the costs associated with these methods justify the added value and that the results are consistent with farmers’ own experiences and observations. In other words, all “universal knowledge” needs to be localised to the farmer’s specific setting and integrated into different farming domains and processes.

In future approaches to reduce P losses, all stakeholders, including the research community, authorities and farmers, need to develop insights into the specificity of farming systems and their dynamic relations with local conditions (Stuiver et al. 2004). These local conditions may relate to soil P content, farm manure production, soil sorption capacity or vulnerability to erosion or overland flow, among others. Farmers’ knowledge is defined as their capability to coordinate and (re-)shape a wide range of socio-technical growth factors within specific locations and networks towards desired outcomes (Stuiver et al. 2004), such as sustainable production and/or reduction of nutrient losses. Implementation of abatement measures needs to be thoroughly discussed, shaped and adjusted to specific local conditions, and this is possible only by embracing farmers’ local knowledge.

To study the relative effectiveness of different methods for identification of CSAs and P losses from arable fields, a study was performed in Sweden on a set of 16 demonstration farms. Researchers in cooperation with farmers on the 16 farms performed and evaluated (i) soil sampling based on high-resolution erosion modelling and consequent analyses of soil chemical properties, (ii) independent risk assessments by farmers compared with the results of high-resolution overland flow and erosion modelling and (iii) SWOT (strengths, weakness, opportunities and threats) analysis performed by the farmers. The first section of the paper describes the characteristics of the study farms and how the methods described above were applied. The following section presents the results from the study followed by a discussion of these results and their relevance for identifying effective abatement measures.

## Materials and methods

### Study farms

**Fig. 1:**
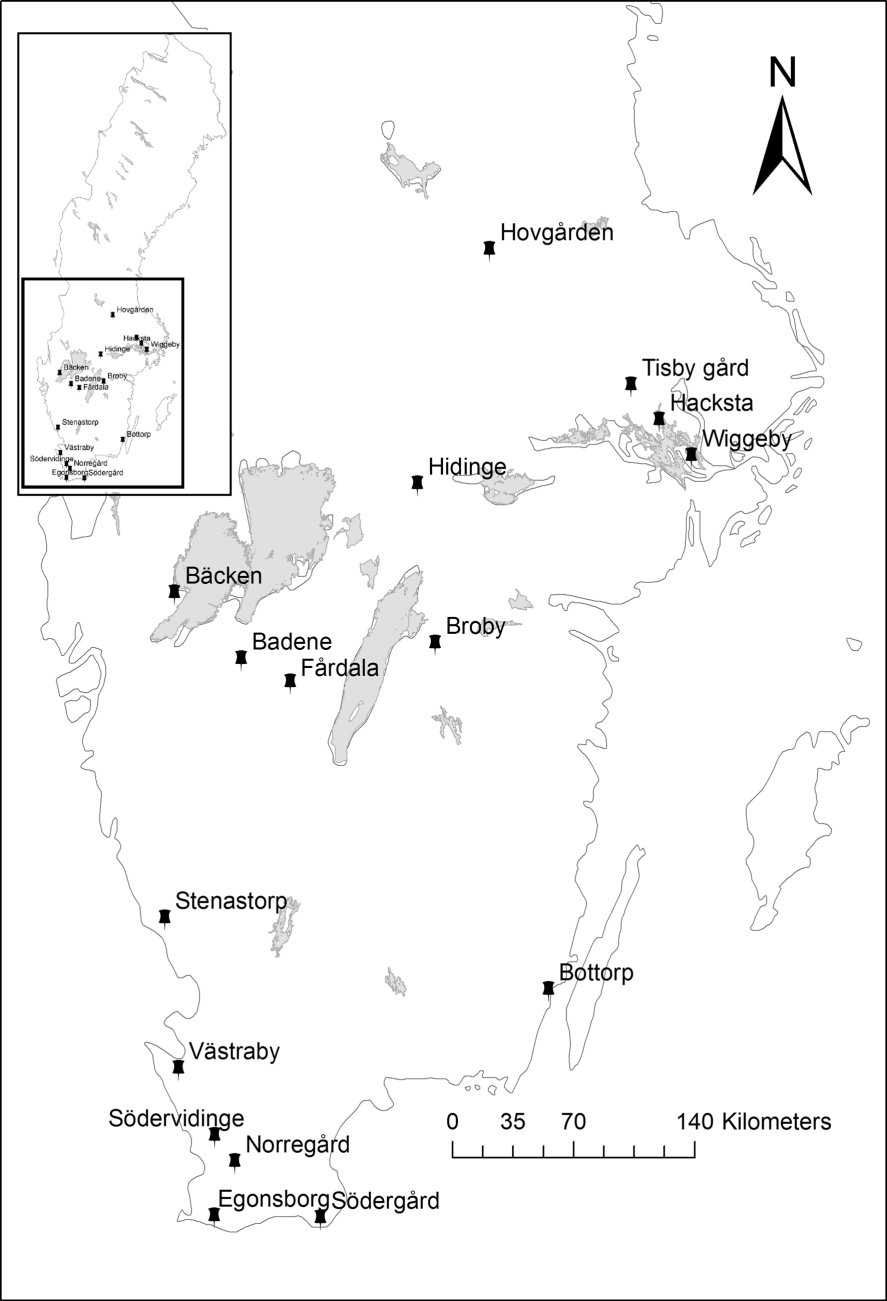
Southern Sweden with the location of the 16 farms included in this study

The farms selected for the study were 16 demonstration farms included in the project *Farming in Balance* (FiB, http://www.odlingibalans.com/). These farms are located in arable areas of southern and central Sweden stretching from Skåne in the south to Dalarna in the north (Fig. 1). The characteristics of each farm are summarised in Table 1. They cover a wide range of climate, pedological, hydrological and production conditions. These particular farms within the FiB project are intended to serve as a bridge between research and practical farming, making them very suitable as study areas for the present analysis. Data on all fields and parcels belonging to each farm were downloaded as GIS vector layers from the Swedish Board of Agriculture database in the form of agricultural blocks. These blocks were then imported to Google Earth to create high-resolution images of each farm. These images were then sent to the 16 farmers for identification of CSAs based on their own experience and observations.

**Table 1:** Characteristics of the 16 farms included in this study

Farm	County	Production	Area (ha)	Soil texture	Temperature^a^ (°C)	Precipitation^a^ (mm)
Egonsborg	Skåne	Crop production	450	Sandy loam, sandy clay loam	8.5	698
Löderop	Skåne	Crop production, pig and beef	165	Loam, sandy loam	8.0	734
Norregård	Skåne	Crop production	90	Loam, sandy loam	8.2	783
Södervidinge	Halland	Crop production, vegetables	135	Loam, sandy loam	8.5	741
Västraby	Skåne	Crop production and dairy	650	Sandy clay loam, clay loam	8.4	725
Bottorp	Kalmar	Crop production and chickens	411	Sandy clay loam, clay loam	7.6	565
Stenastorp	Halland	Crop production	58	Sandy loam	7.6	1026
Fårdala	Västra Götaland	Crop production and dairy	160	Sandy loam, loam	6.2	785
Badene	Västra Götaland	Crop production and pigs	237	Silty clay, clay	6.9	688
Broby	Östergötland	Crop production and hens	320	Sandy loam, clay loam	6.8	603
Bäcken	Västra Götaland	Crop production and pigs	670	Silty clay loam, silty clay	6.9	777
Hidinge	Örebro	Crop production and pigs	180	Silty clay, silty clay loam	6.0	784
Wiggeby	Stockholm	Crop production	600	Clay, clay loam	6.9	586
Hacksta	Uppsala	Crop production and grazing animals	350	Clay, silty clay	6.5	586
Tisby	Uppsala	Crop production	168	Silty clay, clay	6.4	611
Hovgården	Dalarna	Crop production, pigs and beef	330	Silt loam, silt	5.3	670

^a^Mean annual values 1981–2011 from Swedish Meteorological and Hydrological Institute (http://luftweb.smhi.se/)

### Soil sampling and analyses

In order to quantify potential P mobilisation from the most vulnerable parts of the fields, soil sampling was carried out in the vulnerable areas identified through distributed modelling with high-resolution DEM performed for this study (see Section “Modelling and farmer evaluation of overland flow and erosion” below for a detailed description of the modelling process). A total of 163 soil samples were collected in the immediate vicinity of the modelled erosion pathways, identified as the top 2% of all 2m × 2 m cells with the highest erosion values on each of the 16 farms. The selection of sampling points was also based on soil maps in order to cover different soil textural classes. Each sample (10 cm deep) consisted of 15 soil cores collected from an area of 1 m^2^. These soil samples were air-dried, gradually broken down by hand and sieved (<5 mm) before analysis with DESPRAL test, and the content of plant-available P was determined by extraction with ammonium lactate/acetic acid (P-AL) at pH 3.75 (Egnér et al. 1960). The risk of sediment, dissolved and particle-bound P mobilisation was estimated with the DESPRAL test, performed as described by Withers et al. (2007), who showed that the results of the DESPRAL test correlated well (*r* ^2^ = 0.7–0.8) with the amounts of SS, total P and dissolved P in overland flow generated by indoor simulated rainfall. DESPRAL is an environmental soil test developed to estimate the intrinsic risk of sediment and P mobilisation from agricultural soils, where both dispersed particles and P are simultaneously quantified (Villa et al. 2014). Suspended solids (SS), total P (TP) and dissolved P (DP) were determined in DESPRAL aliquots in accordance with the methods issued by the European Committee for Standardization (European Committee for Standardization 1996). Suspended solids were determined by filtration through 0.2-μm pore membrane filters dried at 105 °C while turbidity, which is highly correlated to SS (Villa et al. 2014), was measured on post-dispersion aliquots using a Hach 2100AN instrument (Hach Company, CO) and expressed as nephelometric turbidity units (NTU). Total phosphorus was determined colorimetrically after digestion of unfiltered samples in acid persulphate solution, DP was determined on filtered samples (0.2-μm pore membrane filters) using Gallery Plus Photometric Analyzer Thermo Fisher. Unreactive P (UP) was calculated as the difference between TP and DP.

Plant-available soil P, potassium (K), calcium (Ca) and magnesium (Mg) concentrations were determined by extraction with ammonium lactate/acetic acid (P-AL) at pH 3.75 (Egnér et al. 1960), which is the standard agronomic soil P test used in Sweden. The same extraction was also used to analyse iron (Fe) and aluminium (Al) as indicators of soil P sorption capacity (Ulén 2006). Simple and multiple regression analyses were performed to study relationships between the results from AL analyses and DESPRAL tests. One-way analysis of variance (ANOVA) with a Fisher comparison test was used to test differences between P-AL classes and soil textural classes regarding DP and UP release in DESPRAL tests. All statistical analyses were performed using Minitab 16.1.1.

### Modelling and farmer evaluation of overland flow and erosion

All modelling simulations were performed prior to the farmers’ self-evaluations as described in Djodjic and Villa (2015). The base layer for the modelling work was a DEM in raster format. A 2-m grid based on LiDAR data was used, with a density of 0.5–1 point m^−2^ and accuracy which is usually better than 0.1 m (Lantmäteriet 2014). The modified USPED model (Mitasova et al. 2001) was implemented within a frame of PCRaster software for environmental modelling (Schmitz et al. 2009). In brief, USPED is a simple model which predicts the spatial distribution of erosion and deposition patterns based on the change in overland flow depth and on the local geometry of terrain, including both profile and tangential curvatures. The slope length factor (LS) of the RUSLE equation is replaced with upslope contributing area in the modified model and the LS factor is calculated using

1 $$ {\text{LS}} = \left( {\frac{A}{22.13}} \right)^{1.6 } \cdot\,(\sin b)^{1.3}, $$

where *A* is the upslope contributing area and *b* is the slope angle. Exponent values of 1.6 and 1.3 were used here, as recommended by Mitasova et al. (2001). In the modified USPED, the erosivity factor (*R*), soil erodibility factor (*K*) and vegetation cover factor (*C*) were used in accordance with the following: the catchment-specific mean annual runoff (Table 1) was used as the rainfall erosivity factor (*R*), the values of soil erodibility factor (*K*) were based on the new soil map of textural classes of Swedish agricultural soils (Paulsson et al. 2015), in combination with soil maps from the Geological Survey of Sweden for non-agricultural areas, and each soil textural class was assigned a specific *K* value according to Stone and Hilborn (2012). Land use map and cover factor (*C*) values from Stone and Hilborn (2012) were combined to spatially distribute the effects of vegetation cover. Since the aim of the modelling was to compare and rank relative long-term erosion and overland flow risk, all arable soil was assigned the same cover factor (*C*) representative for cereal crops (*C* = 0.35), without consideration for the actual crop distribution. In order to separate and better visualise the sub-areas of agricultural land most prone to overland flow, erosion and water ponding, the results obtained in erosion modelling were post-processed as described in Djodjic and Villa (2015). In short, using the “Slice” tool with the “Equal Area” method and 50 output zones within ArcGIS 10.2.1 (©1999-2013 Esri Inc.), 2-m grid cells were reclassified and ranked according to modelled erosion vulnerability. This approach allows incremental identification of CSAs starting with the top 2% of total agricultural area with the highest erosion values according to modelling results and thereafter, if necessary, stepwise 2% increases.

A meeting was organised to present modelling results and allow farmers to work with self-evaluations of CSA risk based on their own knowledge of their fields. At the meeting, the farmers were first given a short introduction and examples of how to consider and report (describe and draw on the map) different types of CSAs, such as frequent overland flow pathways, erosion channels and routes, frequent occurrence of flooding and ponding water on the fields, inadequate drainage and compacted soils. All observations drawn on maps by farmers were then digitised for comparison with the modelled values.

It should be stressed that the modelling work was completed prior to farmers’ self-evaluation. After the self-evaluations were completed, the model results were then compared with the self-evaluations directly at the same meeting. No calibration of the model or its parameters was made to achieve a possible better fit with farmer observations. The abovementioned 2% top-ranked cells were then compared against the CSAs identified by farmers, using the “Selection by location” tool within ArcGIS 10.2.1 (©1999-2013 Esri Inc.), which identified all observed areas that intersected with the modelled areas. The comparison was discussed with farmers and farmers’ reactions and impressions were documented.

### SWOT analysis

At the same meeting mentioned above, all farmers included in the FiB project (*n* = 16) were asked to perform a SWOT (strengths, weakness, opportunities and threats) analysis (Learned et al. 1969) to assess not only the internal strengths and weaknesses of their farm, but also external opportunities and threats (Grošelj and Zadnik Stirn 2015). The SWOT analysis is widely applied in strategic decision support for business management, but recently it has also been used for environmental management and assessment (Scolozzi et al. 2014). Prior to the SWOT analysis, the farmers were introduced to the concept of P index (Lemunyon and Gilbert 1993), where factors governing P losses were grouped into two categories: P sources and P transport pathways. The farmers were asked to categorise their statements in the SWOT analysis into one of these two categories or a third category (Other).

## Results

### Soil sampling and analyses

**Fig. 2:**
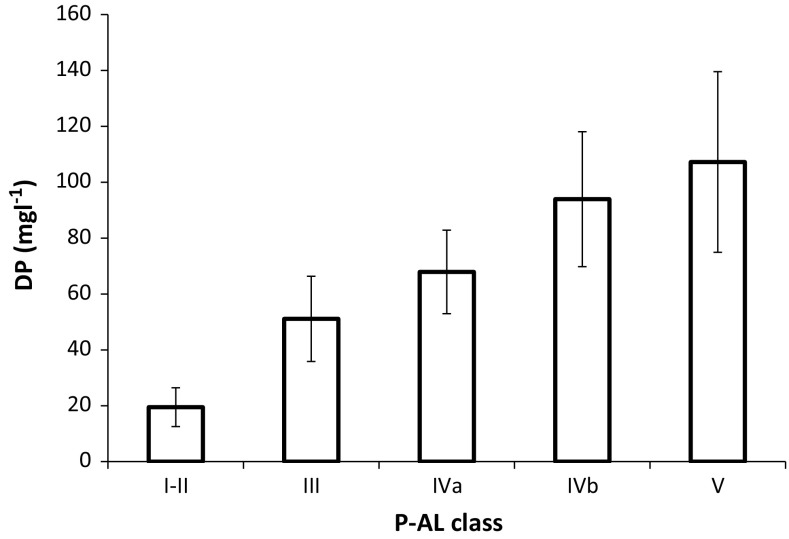
Mean concentration (mg L^−1^) of dissolved P (DP) released in the DESPRAL test as a function of soil test P class (P-AL class). The two lowest classes (I and II) are considered to represent P-deficient soils, class III is considered the optimum P class, whereas the three highest classes (IVa, IVb and V) represent soils with an excessive P content that may pose higher risks for losses to aquatic environments. The bars represent standard deviation

The results from the 163 individual samples included in this study showed a highly significant (*p* < 0.001) but rather weak (*r* ^2^ = 0.28) relationship between P-AL and DP in the DESPRAL test, suggesting a positive, but rather diffuse, relationship between P-AL and DP release. The relationship between P-AL and TP in the DESPRAL test was not statistically significant (*p* = 0.114). Inclusion of soil sorption properties approximated with Al and Fe content served to strengthen the relationship with DP. Thus, the degree of P saturation (DPS), calculated as the ratio between P-AL and the sum of Al-AL and Fe-AL (calculated on a molar basis), showed a highly significant (*p* < 0.001) and stronger (*r* ^2^ = 0.39) relationship with DP in the DESPRAL test. Similarly, ANOVA analysis and the Fisher comparison test showed significant differences between P-AL classes and DP in the DESPRAL test (Fig. 2). All differences were significant with the exception of classes IVb and V (I–II < III < IVa > IVb = V).

The potential mobilisation of UP, determined with the DESPRAL test, was significantly and positively correlated with mobilisation of soil particles. Thus, there was a significant positive relationship between UP concentration and both turbidity (*p* < 0.001, *r* ^2^ = 0.62) and SS (*p* < 0.001, *r* ^2^ = 0.56). Turbidity and SS were also strongly correlated (*p* < 0.001, *r* ^2^ = 0.71), with higher SS concentrations in relation to turbidity for silty soils and higher turbidity in relation to SS concentrations for clayey soils.

The ANOVA results revealed significant differences between soil textural classes in potential mobilisation of both soil particles and UP, with significantly lower concentrations of SS and UP mobilised in sandy soils (loamy sand, sandy loam, sandy clay loam and loam) (Table 2). This was also true for turbidity. The patterns were not as clear for soils with higher clay content, although there was a tendency for soils with a high content of clay (clay) and silt (silt, silt loam and silty clay) to show higher potential mobilisation. Illustrating soil erosion and UP loss vulnerability with a simple soil dispersion test was appreciated by farmers as condensing general, rather abstract knowledge to simple, understandable indicators of water quality (high turbidity = high SS = high UP).

**Table 2:** Analysis of variance (ANOVA) table for different soil textural classes regarding turbidity, suspended solids (SS) and unreactive phosphorus (UP). *N* = number of samples, values are means, with standard deviation in brackets. *Different capital letters* indicate significant difference between soil textural classes (*p* < 0.001)

Soil	*N*	Turbidity (FNU)				SS (mg L^−1^)						UP (mg L^−1^)			
Loamy sand	4	211 (140)			C	272 (136)					H	865.5 (336)		J	K
Sandy loam	39	353 (200)			C	513 (266)					H	772.4 (291)			K
Sandy clay loam	8	372 (128)			C	557 (226)					H	744.6 (182)			K
Loam	24	402 (199)			C	543 (287)					H	884.8 (378)			K
Clay loam	12	1093 (664)		B		1021 (430)				G		1151.7 (240)	I	J	
Silty clay loam	11	1408 (862)	A	B		1519 (709)			F			1169.5 (419)	I	J	
Silt	7	1426 (307)	A	B		2779 (1083)	D					1425.9 (307)	I		
Silt loam	12	1584 (803)	A			2041 (834)		E				1384.3 (439)	I		
Silty clay	19	1446 (499)	A			1473 (433)			F			1270.7 (343)	I		
Clay	27	1514 (533)	A			1378 (286)			F			1255 (297)	I		

* *FNU Formazin Nephelometric Unit*

### Modelling and farmer evaluation of overland flow and erosion

**Fig. 3:**
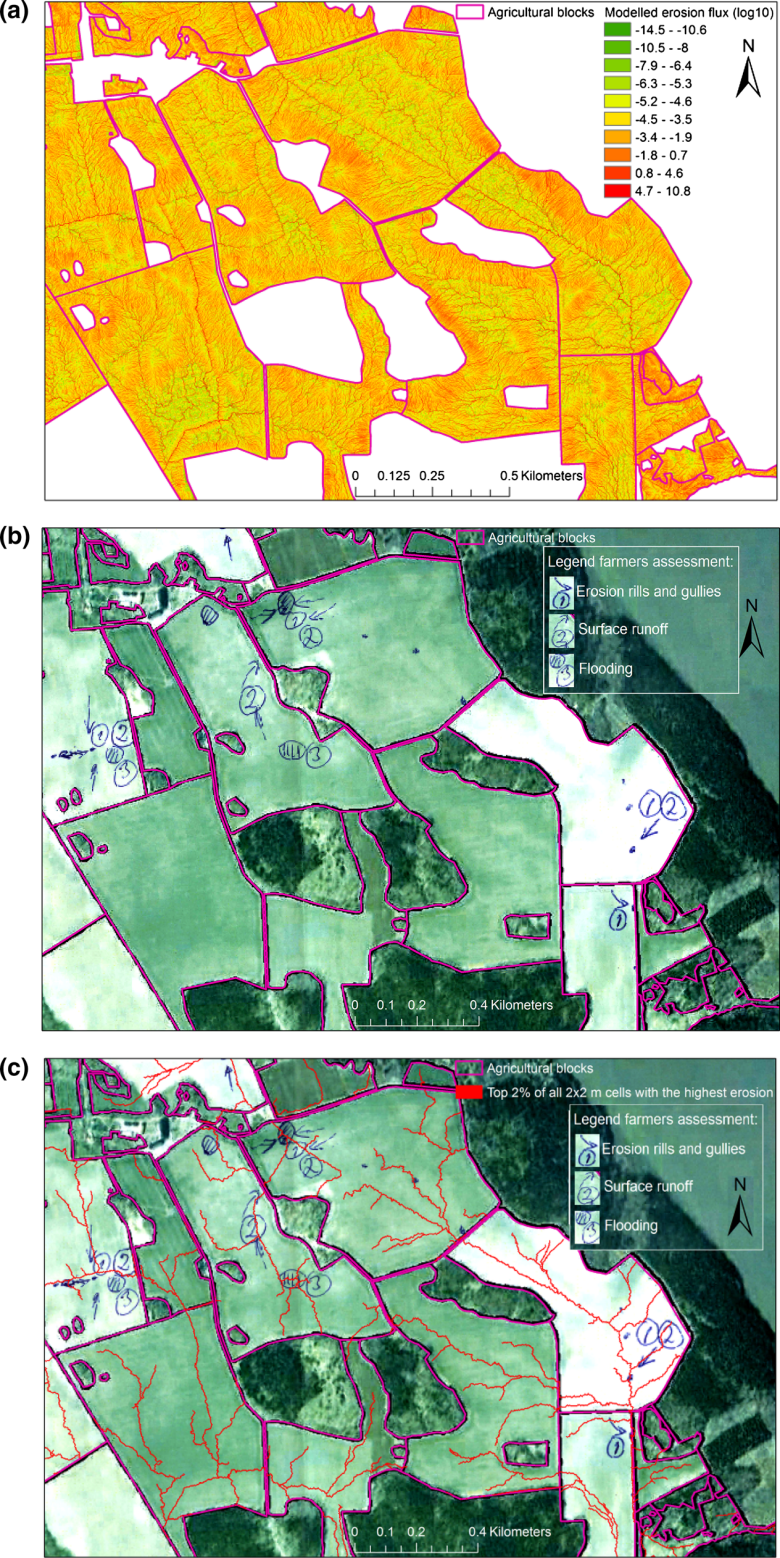
**a** Modelled erosion values, **b** problem areas identified by farmers and drawn on Google Earth map, including erosion, surface runoff and flooding-prone area and **c** top 2% of all 2m × 2 m cells with the highest erosion values (*red lines*) superimposed on farmers’ map of problem areas

Farmers listed and sketched a total of 128 problematic areas in their fields. The different types of problem areas identified are listed in Table 3. The average area of these areas was 1.8 ha, but with wide variations (range 0.024–35.3 ha), emphasising the spatial variability of these features in the landscape. Spatial comparison of observed and modelled features showed that the top 2% of all 2m × 2m cells with the highest modelled erosion values intersected 109 of the 128 (85%) problem areas identified by farmers. An example showing modelling results and farmer evaluation is given in Fig. 3. This is comparable with the findings in earlier similar studies, where Djodjic and Villa (2015) were able with a similar modelling approach to identify 72–96% of observed erosion and overland flow features based on field surveys in four different catchments. Four out of 10 flooded areas (Table 3) not identified by the model were described by farmers as having problems with tile drains. Further, the model was unable to identify most of the CSAs where overland flow and erosion were caused by tramlines and compacted soil.

**Table 3:** Summary of comparison between farmers’ own assessment of specific problem areas on their farm and areas identified by erosion and overland flow modelling

Problem	Number of areas identified by farmers	Number of farmers’ observations identified by model
Overland flow/erosion	38	36
Flooding, drainage problems	72	62
Soil compaction, wheel tracks	8	6
High slope	7	4
Other	3	1
Total	128	109

### SWOT analysis

**Fig. 4:**
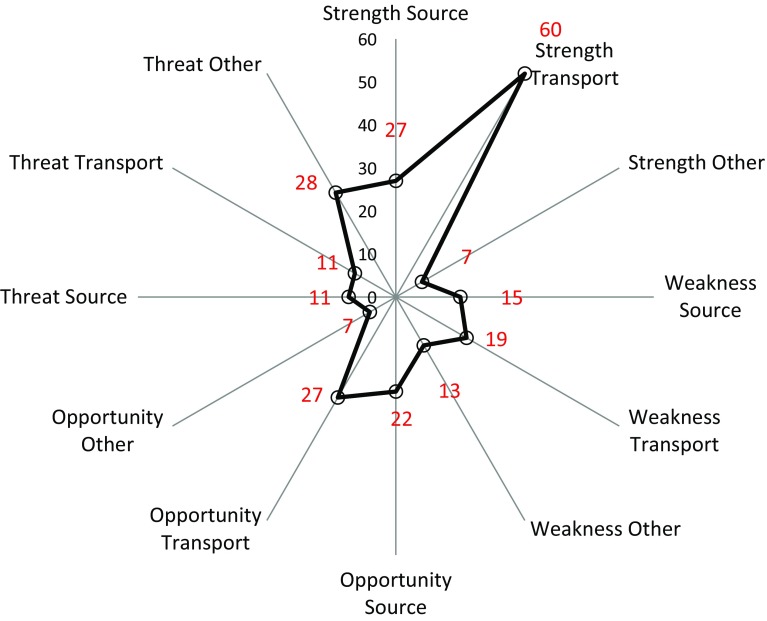
Summary of the farmers’ SWOT (strengths, weaknesses, opportunities, threats) analysis. Numbers reflect the number of statements grouped into SWOT categories, divided into the three P-index subcategories (sources, transport pathways and other)

The result of farmers’ SWOT analyses is shown in Fig. 4. Most observations were categorised as strengths (94 in total), with the focus on transport strength (60) followed by source strength (27). Examples of transport strength were well-drained soil with stable soil structure, functional crop rotation with leys and low or no occurrence of overland flow and erosion. Examples of source strengths were optimal P-AL content (P-AL class III), balanced P management and fertilisation, and well-developed trade in manure to neighbours. Weaknesses (47) and opportunities (56) were evenly distributed between sources and transport pathways. High soil P content, high animal density and manure production were frequently listed as source weaknesses (15 statements). Transport pathway weaknesses identified (19 statements) included erosion and overland flow vulnerability, inadequate drainage and flooding. Most of the opportunities listed directly addressed observed weaknesses, with specific countermeasures based on specific weaknesses, showing farmers’ valuable skills, knowledge and flexibility in finding appropriate countermeasures. For example, optimised placement of buffer strips was suggested to prevent and counteract erosion and overland flow, investment in tile drainage was raised as a method to manage flooding, and manure trading and neighbour collaboration were proposed to solve a local surplus of manure.

Farmers indicated relatively few threats regarding both sources (11) and transport pathways (11). Most threats were listed in the group Others (28) and addressed external effects on farm profitability and possible enforcement of compulsory buffer strips and set-aside, but also included fertiliser taxes, more rigorous animal density rules and broadening drinking water protection areas. Farmers also mentioned the negative effects of climate change with uneven precipitation as a possible future threat.

## Discussion

Previous findings that high-resolution environmental data allow reliable identification of CSAs at the catchment level (Djodjic and Spännar 2012; Djodjic and Villa 2015) are consistent with the results of this study at the farm level. While the study estimate of the high intersection between farmers’ observations and modelled lines reported above is technically correct, it is however difficult to interpret it in terms of goodness of fit. Farmers used different symbols (such as arrows and/or polygons, as in Fig. 3) and sizes to illustrate CSAs. Therefore, while presenting the validity of the modelling results compared with farmers’ experience-based observations in statistical terms might be weak, the following quotes give an indication of the farmers’ own judgement of the goodness of fit:

Modelling results are in very good agreement with my observation of ponded fields (Västraby farm),

Model was accurate in identifying risk areas (Hidinge farm),

Results from the model are useful in daily drift (Bottorp farm),

Modelled maps are not lying (Fårdala farm),

The modelled red lines are ‘dead on target’! (Norregård farm).

The general impression was that the two separately conducted assessments were complementary and can be used to identify CSAs. Farmers’ observations can be used both to confirm/reject modelled results and to better delimit areas of visible impact. Modelling results which coincide well with farmers’ own observations and experience can strengthen farmers’ knowledge, motivate them to target their problem areas and give them valuable data support in discussions with authorities. The model uses flow accumulation as an important factor, where especially convergent flow pathways are recognised, and consideration of the top 2% of all cells with the largest modelled erosion created line features in the landscape which successfully encompassed the observed features identified by farmers, but also extended beyond these observed features, both up- and downslope. Pionke et al. (1997) viewed catchments as “a collection of P sources, storages and sinks tied together by a flow framework” and that “the interaction between P sources, storages and sinks, and flow pathways defined the key linkages from source to impact area”. In that regard, the continuous modelled red lines in Fig. 3c may provide insights into landscape connectivity and help identify the causes behind visible points of impact. Since the C factor was kept constant for all arable land, the modelled results are mostly influenced by and sensitive to topography (i.e. LS factor) and soil erodibility (K factor). The small-scale, in-field spatial variability is driven by topography, whereas modelled differences in erosion levels between fields and farms are governed also by soil distribution.

The present study also showed that farmers usually have very good knowledge on the spatial distribution of problem areas on their own farms. From farmers’ perspectives, the research-modelled results serve rather as a confirmation of their own observations and give relevance to their knowledge and experience. Analytical results from the soil samples collected in this study proved to be useful in several ways. As mentioned above, the farmers who participated in the study were well aware of the importance of P-AL values in the assessment of P loss risks. In the SWOT analysis, high amounts of manure and fields with high P-AL were both assessed as a weakness/threat, while a balanced fertilisation strategy was listed as strength and/or opportunity. However, there were several doubts and questions regarding the reliability of P-AL as an environmental indicator for assessment of the P loss risk. In this context, the DESPRAL test proved to be a useful approximation of DP release. The positive relationship between P-AL and DP in the DESPRAL test illustrated the importance of soil P content for P release. On the other hand, analysis of soil P binding capacity was new and appreciated information for farmers. In addition, concentrations of Al and Fe in AL extract as indicators of P sorption capacity raised questions about the importance of soil pH for both P solubility in general and the results of the P-AL extraction in particular. The acid AL extraction tends to overestimate levels of plant-available P (P-AL) in calcareous soils with high pH (Lovang 2015). However, exemplification of results and relationships based on their own soil samples increased farmers’ willingness both to accept cause–effect reasoning and to consider abatement measures.

Assessment of the potential mobilisation of soil particles and particulate P with the DESPRAL test confirmed earlier results regarding the vulnerability of different soils to erosion (Villa 2014), with a rather clear pattern with lower levels of mobilised particles for lighter soils [(loamy sand, sandy loam, sandy clay loam, loam), Table 2]. The UP concentrations showed a strong correlation with both turbidity and SS levels, meaning lower potential mobilisation of UP in lighter soils. The multiple regression showed that P-AL values affected the levels of potentially mobilised PP, but the UP levels were mainly dependent on the mobilisation of soil particles. Thus, it is important to emphasise that the level of UP losses is controlled to a lower degree by soil P concentration and is more dependent on soil vulnerability to erosion. Combined identification of hydrologically active parts of the landscape (USPED modelling) and the mobilisation vulnerability of soil particles and P (DESPRAL test) creates a reliable basis for decision making by farmers. Once the results prove to be in agreement with farmers’ own assessments, observations and experience, this creates a much needed stimulus to develop locally optimised measures that can be integrated with other processes on the farm. Future model development should focus on the introduction of dynamic modelling and quantification of the losses and transports of both SS and P. Such development would allow for comparisons of modelled results with the measurements of water quality within monitoring programmes.

Although there is some overlap, the results of this project indicate two main groups of farms with differing opportunities for reducing P losses:

1. *Farms with lighter, well*-*drained sandy soils* The main problem in this group is usually linked to P sources, with high P-AL levels in the soil and/or high animal density and manure application rates, which lead to high P losses, primarily in the form of DP. The main focus on such farms should be to optimise manure management and embrace “4R” nutrient management (right form, right time, right place, right amount; Sharpley et al. 2015) to enable optimum fertilisation based on crop demands, thus eventually reducing high soil P-AL concentrations. Two key uncertainties for these farms are the lack of data on soil P binding capacity and the suitability of P-AL as a method for assessment of plant-available P and P release. Abatement options on these farms should focus on the stepwise reduction of P sources (optimised fertilisation, manure trade, cooperation with neighbours to reduce manure surpluses) and purification of water leaving the fields. Manure application should be performed under optimal conditions, followed quickly by incorporation. The question is whether ordinary wetlands and P sedimentation ponds would be effective, since they mainly reduce UP losses. Ponds and wetlands with subsequent chemical water purification (Ekstrand et al. 2011) could be an effective measure in this case. General buffer strips and structure liming would have very limited effect in these soils and should not be recommended. Optimised buffer zones may be effective in relatively small parts of fields experiencing problems with surface runoff, erosion and flooding.
2. *Farms with heavier soils with higher clay content* The main problem for this group is usually connected to P transport pathways, where the topography and poor drainage lead to overland flow and erosion, with UP as the main form of P. The main focus for these farms should be on identifying and addressing hydrologically active parts of the landscape. The lack of data on soil erosion vulnerability could be solved by DESPRAL analysis. Structure liming (amendment of quicklime or slaked lime to clay soils to improve soil stability, aggregate strength and porosity), optimised buffer zones, grassland farming, adjusted crop rotation and improved drainage are adequate measures for reducing UP losses in these cases. Wetlands and P dams may also reduce levels of UP in water leaving the farm. Well-functioning ditches between forest and arable land can help prevent water flowing from forest over to farmland and reduce runoff-driven mobilisation of P-rich small particles. It is important to know that high P losses can arise from hydrologically active parts of the landscape, even if the P content in the soil is low or moderate (Villa et al. 2014; Djodjic and Villa 2015).

The results of the SWOT analysis in this study showed that farmers recognise both strengths and opportunities on their farms. They are also well aware of the weaknesses and in most cases have suggestions for creative solutions to their problems, with observed weaknesses addressed with proposals in opportunities. A vast majority of the statements made on strengths and opportunities were of an internal character and only in few cases linked to external actors, such as ongoing (strength) or potential (opportunity) cooperation with neighbours and a good relationship with the authorities. On the other hand, farmers viewed the majority of threats as external, with the most prominent threats mentioned being reduced profitability due to a possible introduction of new mandatory rules (e.g. mandatory buffer zones), new restrictions (lower stocking density, forbidding manure spreading) or taxes (fertiliser tax).

Unfortunately, farmers’ knowledge and skills are not utilised today in the development of measures to reduce nutrient losses. According to the farmers in this study, it is currently permissible to apply some general measures (e.g. buffer strips) in fields where these are redundant and will not contribute to the diffuse pollution mitigation. Examples of this are the introduction of wide buffer strips on permeable, well-drained, organic soils and sandy soils, embanked fields and generally fields where overland flow and erosion never occur. Such measures are ineffective, costly for society and frustrating for farmers.

## Conclusions

The high spatial variation in nutrient transport processes demands spatial adjustment of the placement and extent of measures to reduce nutrient losses, in order to maximise their efficiency. This study, based on 16 farms across southern and central Sweden, showed that a combination of farmers’ risk assessments, soil survey and analyses, and high-resolution distributed modelling can successfully identify areas prone to P losses. However, the main lesson of this work covering 16 real-world farms is that it is difficult to find a “one-size-fits-all” method to identify CSAs due to a wide range of factors governing P losses. Consequently, the same is true for abatement strategies which have to be adjusted to local preconditions, transport pathways and P forms. Significant statistical differences in DP release were found between soil P test classes, whereas soil textural classes and not P content governed potential mobilisation of soil particles and UP. Performed SWOT analysis indicates that modern farmers are well-educated, skilled entrepreneurs willing and able to take into account different aspects of their activities, but that additional farm-specific data and information are usually needed to convince and motivate them to develop and implement site-specific abatement strategies based on local conditions. The farmers included in this study unanimously agreed that countermeasures that are in line with farmers’ own understanding of their needs and effects would result in greater acceptance and therefore higher implementation rates than those currently achieved. From a standpoint of communication between farmers, authorities and researchers, risk maps and simple soil tests are very useful tools both to achieve a common understanding of the problem and to make prioritisations regarding selection and spatial placement of countermeasures. In this regard, even the rather coarse classification of farms into the proposed two groups based on probable causes of high P losses (surface runoff/erosion on heavier soils or loss of dissolved P from sandy soils due to high soil P status and manure surplus) was for farmers an important distinction and an indication of where to put their abatement efforts.

Risk maps with identified pathways of overland flow and erosion such as those in Fig. 3 could be used along with other geographical information (maps of clay content, soil test P, etc.) to implement appropriate countermeasures (buffer strips, constructed wetlands, P ponds, structure liming) with high precision and efficiency. A possible way forward could be to make modelled high-resolution results available to authorities and farm advisors to use in discussions with farmers on the possible placement and selection of appropriate measures that are cost effective and consistent with other activities and operations on their own farm. The availability of high-resolution DEM is increasing and with these data already available for many countries in Northern Europe (Tattari et al. 2012) similar assessments can also be performed outside Sweden as well.
